# Systematic review and meta-analysis of head-to-head trials comparing sulfonylureas and low hypoglycaemic risk antidiabetic drugs

**DOI:** 10.1186/s12902-022-01158-5

**Published:** 2022-10-19

**Authors:** Vallo Volke, Urmeli Katus, Annika Johannson, Karolin Toompere, Keiu Heinla, Kertu Rünkorg, Anneli Uusküla

**Affiliations:** 1 Department of Physiology, Institute of Biomedicine and Translational Medicine, Centre of excellence in Genomics and Translational Medicine, University of Tartu, 19 Ravila Street, 50411 Tartu, Estonia; 2 Endocrinology Unit, Tartu University Hospital, 8 L. Puusepa Street, 51014 Tartu, Estonia; 3 Department of Family Medicine and Public Health, University of Tartu, Tartu, Estonia

**Keywords:** Diabetes mellitus, Hypoglycemic therapy, Sulfonylurea, All-cause mortality, MACE

## Abstract

**Background:**

Safety of sulfonylurea drugs in the treatment of Type 2 Diabetes is still under debate. The aim of this study was to compare the all-cause mortality and cardiovascular adverse events of sulfonylureas and drugs with a low risk for hypoglycaemia in adults with type 2 diabetes.

**Methods:**

Systematic review and meta-analysis of randomised controlled trials. Data sources: MEDLINE (PubMed, OVID), Embase, Cochrane Central Register of Controlled Trials, CINAHL, WOS and Lilacs.

**Study selection:**

Randomised controlled head-to-head trials that compared sulfonylureas with active control with low hypoglycaemic potential in adults (≥ 18 years old) with type 2 diabetes published up to August 2015. The drug classes involved in the analysis were metformin, dipeptidyl peptidase-4 (DPP-4) inhibitors, sodium-glucose co-transporter-2 (SGLT-2) inhibitors and glucagon-like peptide-1 (GLP-1) receptor agonists.

**Outcomes:**

The primary endpoint was all-cause mortality. The secondary endpoints were MACE, cardiovascular events and severe hypoglycaemia. Synthesis of results: Two reviewers checked study eligibility, independently extracted data and assessed quality with disagreements resolved through discussion. We assessed the risk of bias of the included studies using the Cochrane risk of bias tool for randomized trials v2. Pooled odds ratios (ORs) were estimated by using fixed effects model. The study is registered on PROSPERO (26/05/2016 CRD42016038780).

**Results:**

Our final analysis comprised 31 studies (26,204 patients, 11,711 patients given sulfonylureas and 14,493 given comparator drugs). In comparison to drugs with low hypoglycaemic potential, sulfonylureas had higher odds for all-cause mortality (OR 1.32, 95% CI 1.00-1.75), MACE (OR 1.32, 95% CI 1.07–1.61), myocardial infarction (fatal and non-fatal) (OR 1.67, 95% CI 1.17–2.38) and hypoglycaemia (OR 5.24, 95% CI 4.20–6.55). Subsequent sensitivity analysis revealed differences in the effect of sulfonylureas, with an increased risk of all-cause mortality with glipizide but not the other molecules.

**Conclusion:**

Our meta-analysis raises concern about the safety of SUs compared to alternative drugs involved in current analysis. Important differences may exist within the drug class, and glimepiride seems to have best safety profile.

**Supplementary information:**

The online version contains supplementary material available at 10.1186/s12902-022-01158-5.

## Background

Sulfonylureas (SUs) have been available for the treatment of type 2 diabetes for more than 50 years. These drugs are very effective at lowering glucose [1] and have a low cost for the healthcare system. Until now, the drug class is a viable second- or third-line option after metformin in the type 2 diabetes treatment guidelines [2, 3]. However, hypoglycaemia remains the most important and well-known side effect of SUs [4]. Moreover, the safety of the drug class has been questioned for a very long time [5]. With the appearance of new classes of oral diabetes drugs with a lower risk of hypoglycaemia (DPP-4 inhibitors and SGLT-2 inhibitors), the debate about the role of SUs in diabetes management has intensified. Cost is also a concern regarding the new drug classes, especially in the case of GLP-1 receptor agonists (GLP-1 RAs) [6]. However, there is little direct trial evidence that SUs may pose a risk toward cardiovascular events and/or death; so far, only one recent study in a high-risk population has reported a significantly increased risk with glipizide versus metformin [7]. While new drug classes have been studied in dedicated cardiovascular safety trials, we do not have comparable studies with older drugs. Several meta-analyses have been conducted focusing on the safety of SUs with conflicting results. Thus, major cardiovascular events (MACE) have been reported to be increased in SU users [8], while other studies have found no risk [9–11]. Some of the studies have found no effect on mortality with SU use [10, 12], while others have demonstrated increased mortality [8, 9]. To fill the gap in knowledge, we focused our meta-analysis on the studies with an active control group. We included only studies with a comparator diabetes drug with low risk of hypoglycaemia (metformin, DPP-4 inhibitors, SGLT-2 inhibitors and GLP-1 receptor agonists).

The aim of this study was to perform a systematic review and meta-analysis to compare the odds for all-cause mortality and adverse cardiovascular outcomes attributable to the use of sulfonylureas compared with drugs with a low risk for hypoglycaemia in adults living with type 2 diabetes.

## Methods

We conducted a systematic review and meta-analysis to assess the safety of sulfonylureas in the treatment of type 2 diabetes compared to active control with low hypoglycaemic potential in adults (≥ 18 years old) with the focus on all-cause mortality, MACE (defined as the sum of fatal or non-fatal myocardial infarction, fatal or non-fatal stroke, acute coronary syndrome and cardiac failure, MACE component cardiovascular events and severe hypoglycaemia. As the reporting of side effects, cardiovascular side effects in particular [13], is poor in the clinical trials, we have selected all-cause mortality as a principal safety hallmark in our analysis.

### Inclusion and exclusion criteria

Eligible studies included randomized controlled studies where sulfonylureas (monotherapy or combination therapy) were compared to an active control drug with low risk for hypoglycaemia (metformin, DPP-4 inhibitors, SGLT-2 inhibitors and GLP-1 receptor agonists) in the treatment of type 2 diabetes. We did not include the studies with glitazones because of their well-known cardiovascular side effects and due to the limited use of the drug class after safety concerns were raised [14].

Studies with the following characteristics were excluded: participant age below 18 years or pregnant or follow-up time less than 24 weeks.

### Data sources and searches

The current review builds on a previous systematic review [9]. This systematic review included randomized clinical trials with a duration of at least 24 weeks, enrolling patients with type 2 diabetes, comparing sulfonylureas with placebo or active drugs different from other sulfonylureas up to 31 October 2012 [9]. A total of 116 trials fulfilling the inclusion criteria were identified by Monami et al. Twelve of these studies also met the eligibility criteria for the current review. For additional review, we searched MEDLINE (PubMed, OVID), Embase, Cochrane Central Register of Controlled Trials, CINAHL, WOD, and Lilacs from 1 to 2012 to 6 August 2015. Search strategies were adapted for each search engine. In addition, we searched the reference lists of the potentially eligible studies and clinical trial registries (https://clinicaltrials.gov/).

The full electronic search strategy for OVID MEDLINE is presented in Appendix 1.

### Study selection

Teams of two reviewers (VV and UK, AU and AJ, KR and KH) screened articles independently, first by title and abstract, then by full-text, to determine eligibility for final inclusion (including those from the previous review and from the updated search) and recorded the results onto standardized forms of the preformatted Excel file. At each stage of screening any differences between reviewers were discussed, and a consensus decision for eligibility and inclusion was made for all articles. A third reviewer resolved differences between reviewers, if necessary. In cases where multiple publications were associated with the same RCT, a key paper for each RCT was selected, and then the other associated publications were used for supplementary information during the data extraction process. We developed a data extraction sheet (Appendix 2; based on the Cochrane Consumers and Communication Review Group’s data extraction template) [15], pilot-tested it on ten randomly-selected included studies, and refined it accordingly.

### Data collection process

Two reviewers extracted data independently in duplicate onto piloted standardized forms. If multiple publications were associated with a study, we extracted data from the primary publication (assigned as the publication with the most detailed description of the methods and/or the longest follow-up time). Data reported in the primary publication were used in the case of inconsistencies in the publications based on the same source study. Where available, data were also sought from the U.S. National Library of Medicine clinical trials registry. The two reviewers compared the extracted data and resolved differences by discussion. If there was still a discrepancy, a third reviewer adjudicated. We did not contact authors for additional information.

We extracted data for study identifier, study design, setting, population characteristics, intervention and comparator characteristics (incl. data on co-interventions), risk of bias, quantitative outcomes, source of funding and ethics approval, study limitations, and other important comments.

### Risk of bias

We used the Cochrane risk of bias (RoB) tool 2.0 [16] to assess the risk of bias related to the methodological aspects of the included studies, and disagreement at any stage was solved by consensus or arbitration. The items assessed included the method used to generate the sequence of and concealment of randomization and methods for blinding, incomplete and outcome reporting. To summarize the quality of evidence, we assessed bias in five domains (randomisation process, missing outcome data, deviations from intended interventions, measurement of the outcome and selection of the reported results) plus overall risk in bias in order to classify each trial. The overall risk of bias generally corresponded to the worst risk of bias in any of the domains. However, if a study is judged to have ‘some concerns’ about risk of bias for multiple (two or more) domains, it was judged as at high risk of bias overall.

### Publication bias

We examined the potential for publication bias by using a funnel plot and a statistical test for asymmetry. For each funnel plot, we chose a test for asymmetry in accordance with recent recommendations [17], and a p < 0.10 was taken to indicate statistical evidence of asymmetry.

### Outcomes

We extracted (arm-level data) from each included trial (the total number of participants randomized to each intervention). For outcomes, we also extracted the number of participants who (i) died (of any cause) over the course of follow-up and (ii) reported having MACE (defined as the sum of fatal or non-fatal myocardial infarction, fatal or non-fatal stroke, acute coronary syndrome and/or cardiac failure reported as serious adverse effects), MACE component cardiovascular events and severe hypoglycaemia.

Definitions of secondary outcomes (cardiovascular events and severe hypoglycaemia) correspond to those reported in the originally published papers and/or clinical trial registries. If data on particular outcome was not presented in the article or U.S. National Library of Medicine clinical trials registry, trial was not included in this meta-analysis. If numbers randomized were not reported numbers included in the final analyses or number completing study were used instead.

We evaluated the certainty of evidence for main outcomes with the GRADEpro tool [18].

### Statistical analysis

For each study and outcome, we extracted the number of events and study subjects in the comparison groups. We treated comparisons from trials with multiple intervention groups as independent two-arm studies in the pair-wise meta-analyses. To standardize the reporting of our results, we calculated odds ratios and 95% confidence intervals from the number of events and participants in each group for every trial. We used a fixed effects model (Peto method) [19] meta-analysis to assess the effect of sulfonylureas versus low hypoglycaemic potential active drugs on the outcomes of interest.

We assessed statistical heterogeneity by visual inspection of the forest plots and using the Q-test and I² statistic. We interpreted the I² statistic according to the recommended thresholds [20].

We undertook sensitivity analysis to explore whether the results are sensitive to restriction of studies with low RoB. In addition,we conducted additional analyses to explore the influence of both specific sulfonylureas (glipizide, glimepiride, gliclazide, and glibenclamide) and comparison drugs (metformin, SGLT-2 inhibitors, DPP-4 inhibitors and GLP-1 RAs) on the selected outcomes.

Analyses were done using Stata 14.2; StataCorp LLC, Texas, USA. R package ’robvis’ was used to visualize risk of bias [21].

The study is registered on PROSPERO (26/05/2016 CRD42016038780).

## Results

### Study inclusion and characteristics of included studies

**Fig. 1:**
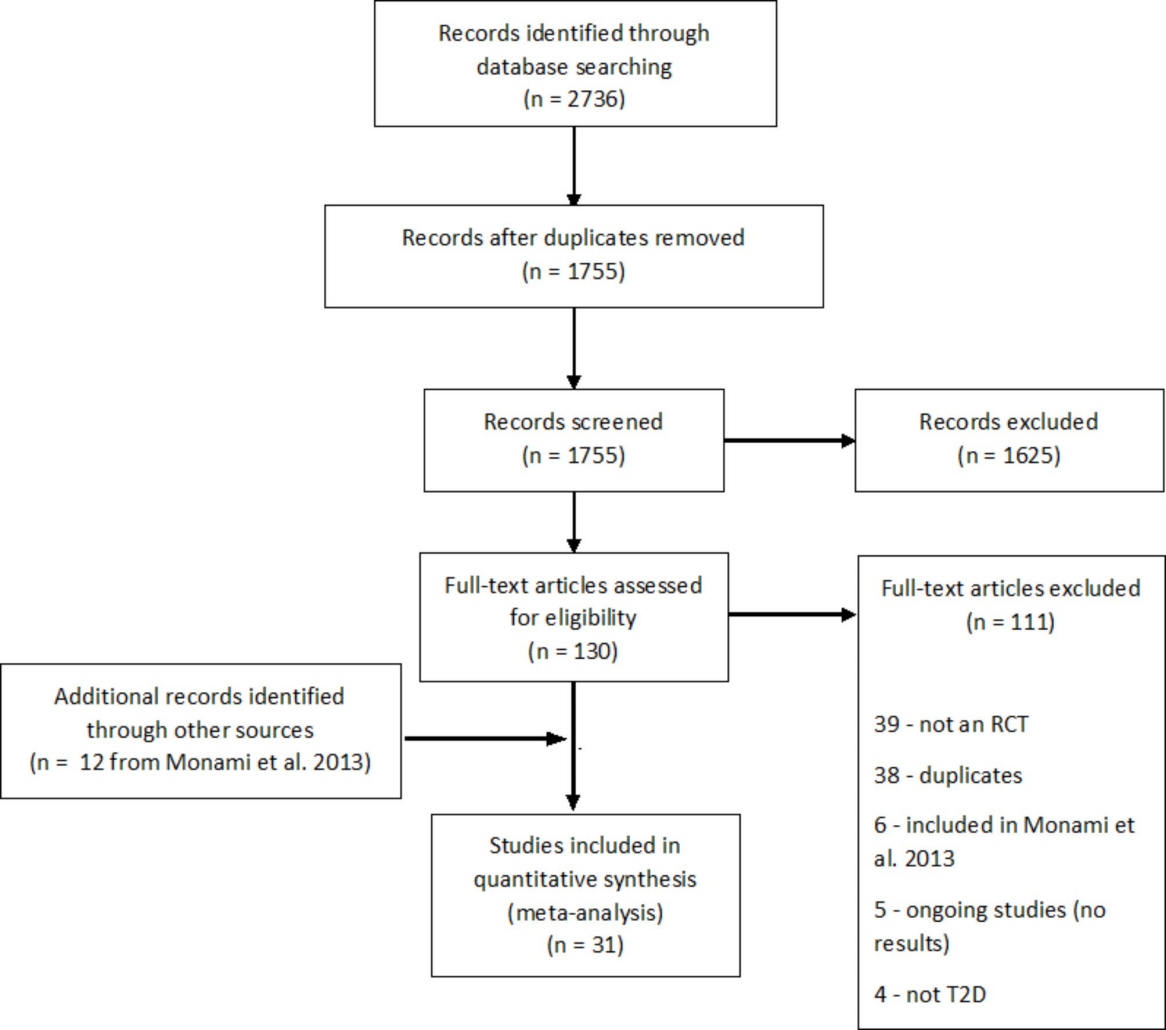
Flow diagram of study identification, inclusion and exclusion

We identified 2736 records through database searching (Fig. 1). From these, 1755 titles and abstracts of were screened, and 130 articles were selected for full-text retrieval, of which 111 were excluded. Reasons for exclusion are described in Fig. 1. This resulted in the inclusion of 19 studies from the database search and 12 studies from the previous systematic review by Monami et al. (Table 1).

**Table 1:** Included randomized clinical trials and their baseline characteristics

Author, year	Number of patients randomized	Trial duration (weeks)	Mean age (years)	HbA1c (%)	BMI	Diabetes duration (years)	Add on (Y-Yes N-No)	Control	SU (ref)
Seck, 2010	1172	104	57	7.3	31.2	5.8	Y Metformin 1.5 g	Sitagliptin 100 mg	Glipizide
									5–20 mg [27]
Seino, 2010	411	24	58	8.9	24.5	8.3	N	Liraglutide 0.9 mg	Glibenclamide 2.5 mg [28]
Arechavaleta, 2011	1035	30	56	7.4	30	6.8	Y Metformin 1.5 g	Sitagliptin 100 mg	Glimepiride
									1–6 mg [29]
Filozof, 2010	1007	52	60	8.5	31	6.6	Y Metformin 1.5 g	Vildagliptin 50 mg twice daily	Gliclazide
									80–320 mg [30]
Foley, 2009	1092	104	55	8.6	30.7	2.2	N	Vildagliptin 50 mg twice daily	Gliclazide
									80–320 mg
									[31]
Garber, 2009	746	52	53	8.3	33.1	5.4	N	Liraglutide 1.2 mg Liraglutide 1.8 mg	Glimepiride
									8 mg [32]
Kahn, 2006	2902	208	57	7.4	32.2	3.7	N	Metformin	Glyburide
		(median)						2 g	7.5 mg twice daily [26]
Ferrannini, 2009	2789	52	57	7.3	31.7	5.7	Y Metformin 1.9 g	Vildagliptin 50 mg twice daily	Glimepiride
									2–6 mg [33]
Yki-Järvinen, 1999	48	52	59	9.8	29.3	NR	Y	Metformin	Glyburide 10.5 mg [34]
							NPH insulin	2 g	
Nomoto, 2016	103	26	61	7.4	25.5	NR	Y	Sitagliptin 100 mg	Glimepiride 0.5-2 mg/day
							55% Metformin		[35]
							Dose NR		
Derosa, 2013	453	52	NR	8.3	27.5	5	Y Metformin 2.2 g Pioglitazone 30 mg	Sitagliptin 100 mg	Glibenclamide 5 mg 3 times a day [36]
Rosenstock, 2013	441	52	70	7.5	29.8	6.1	No	Alogliptin	Glipizide
								25 mg	5–10 mg [37]
Leiter, 2015	1452	104	56	7.8	31	6.6	Y Metformin > 1.5 g	Canagliflozin 100 mg or 300 mg	Glimepiride
									1–8 mg [38]
Erem, 2014	40	52	53	7.9	32.5	NR	N	Metformin	Gliclazide
								2 g Pioglitazone 45 mg	30–120 mg [39]
Ridderstråle, 2014	1549	104	56	7.9	30.2	NR	Y	Empagliflozin 25 mg	Glimepiride 1–4 mg [40]
							Metformin > 1.5 g		
Nauck, 2014	816	104	58	7.7	31.5	6.5	Y, Metformin ≥ 1.5 g	Dapagliflozin 2.5–10 mg	Glipizide
									5–20 mg [41]
Del Prato, 2014	2639	104	55	7.6	31.2	5.5	Y Metformin ≥ 1.5 g or	Alogliptin 12.5 or 25 mg	Glipizide
									5–20 mg [42]
Gudipaty, 2014	47	24	55	6.5	32.3	4	N	Sitagliptin 100 mg	Glimepiride 0.5-4 mg [43]
Hong, 2013	304	156	63	7.6	25.2	5.6	N	Metformin 0.75–1.5 g	Glipizide
									15–30 mg [7]
Pérez, 2015	400	24	49	9.6	24.5	NR	N	Sitagliptin 100 mg	Glimepiride
									2–6 mg [44]
Ferreira, 2013 (1)	129	54	60	7.9	26.8	17.5	N	Sitagliptin	Glipizide
								25 mg	2.5–20 mg [45]
Ferreira, 2013 (2)	426	54	64	7.8	26.8	10.4	N	Sitagliptin	Glipizide
								25 or 50 mg	2.5–20 mg [46]
Schernthaner, 2015	720	52	73	7.6	29.6	7.6	Y Metformin 1.6 g (mean)	Saxagliptin	Glimepiride
								5 mg	1–6 mg [47]
Hartley, 2015	480	32	71	7.8	29.7	8.7	N	Sitagliptin	Glimepiride
								50 or 100 mg	1–6 mg [48]
Gallwitz, 2012	1029	208	56	7.5	32.5	5.7	Y Metformin 2 g (median)	Exenatide	Glimepiride
								10–20 µg	1 mg [49]
Ahrén, 2014	945	104	55	8.1	32.6	6.1	Y Metformin ≥ 1.5 g or MTD	Albiglutide 30–50 mg, Sitagliptin 100 mg	Glimepiride
									2–4 mg [50]
Barnett, 2012	227	52	57	8.1	29.7	NR	N	Linagliptin	Glimepiride
								5 mg	1–4 mg [51]
Nauck, 2013	1091	104	57	8.4	31.1	7.6	Y Metformin 2 g	Liraglutide 0.6, 1.2 or 1.8 mg	Glimepiride
									4 mg [52]
Del Prato, 2015	814	208	58	7.7	31.5	6.4	Y Metformin 1.5–2.5 g	Dapagliflozin 2.5, 5 or 10 mg	Glipizide
									5–20 mg [53]
Göke, 2013	858	104	58	7.7	31.4	5.5	Y Metformin ≥ 1.5 g	Saxagliptin	Glipizide
								5 mg	5–20 mg [54]
Berndt-Zipfel, 2013	44	24	59	7.4	33.9	73	Y	Vildagliptin 100 mg	Glimepiride 0.5-4 mg [55]
							Metformin		
							Dose NR		

NR, not reported

### Study characteristics

All trials used a parallel-group design. The mean sample size was 898 (range: 44 to 4360). In all, 27 trials were multicentric trials (6 to 488 centres) and 4 were single-centre trials. The trials took place worldwide (n = 18) and in single countries (China (n = 2), Japan (n = 2), Italy, the USA, Germany and Turkey). The location was not stated for five trials.

In total, 11 out of 31 trials were multi-arm; six multi-arm trials assessed the same experimental drug at multiple dose levels, seven assessed at least two different drugs and eight assessed both the same experimental drug at multiple dose levels and different drugs. In total, eight trials had a co-intervention, mainly diet and exercise counselling. Only four studies were carried out before the year 2007 (for nine studies the period of data collection was not reported).

Characteristics of the participants: This review included 31 trials, with a total of 26,204 randomized participants. We summarize the characteristics of the participants in Table 1. The mean age of participants was 58.4 years; there were more women (52%) than men (48%).

The comparisons were as follows: 19 trials compared sulfonylureas with DPP-4 inhibitors (n = 14,403); five trials with GLP-1 RAs (n = 3920); four trials with metformin (3294); and four trials with SGLT-2 inhibitors (n = 4631).

### Risk of bias within studies

The risk of bias varied between individual studies, ranging from low to high (Supplementary Figs. 1 and 2). A total of 12 of 31 (39%) RCTs had a low risk of bias, 35% (n = 11) some and 26% (n = 8) a high risk of bias. Most of the studies were sponsored by the pharmaceutical industry. Regarding publication bias, funnel plots suggested no major publication bias (Suppl. Figures 6 and 7; P = 0.34 for all-cause mortality and 0.891 for MACE; Peters test).

### Withdrawals

The overall attrition in studies ranged from 2 to 64%. The differential attrition (the difference in the rates of sample loss for the sulfonylurea and comparator groups of medications with low hypoglycaemic potential) ranged from 11 to 21%.

#### Primary outcomes

**Fig. 2:**
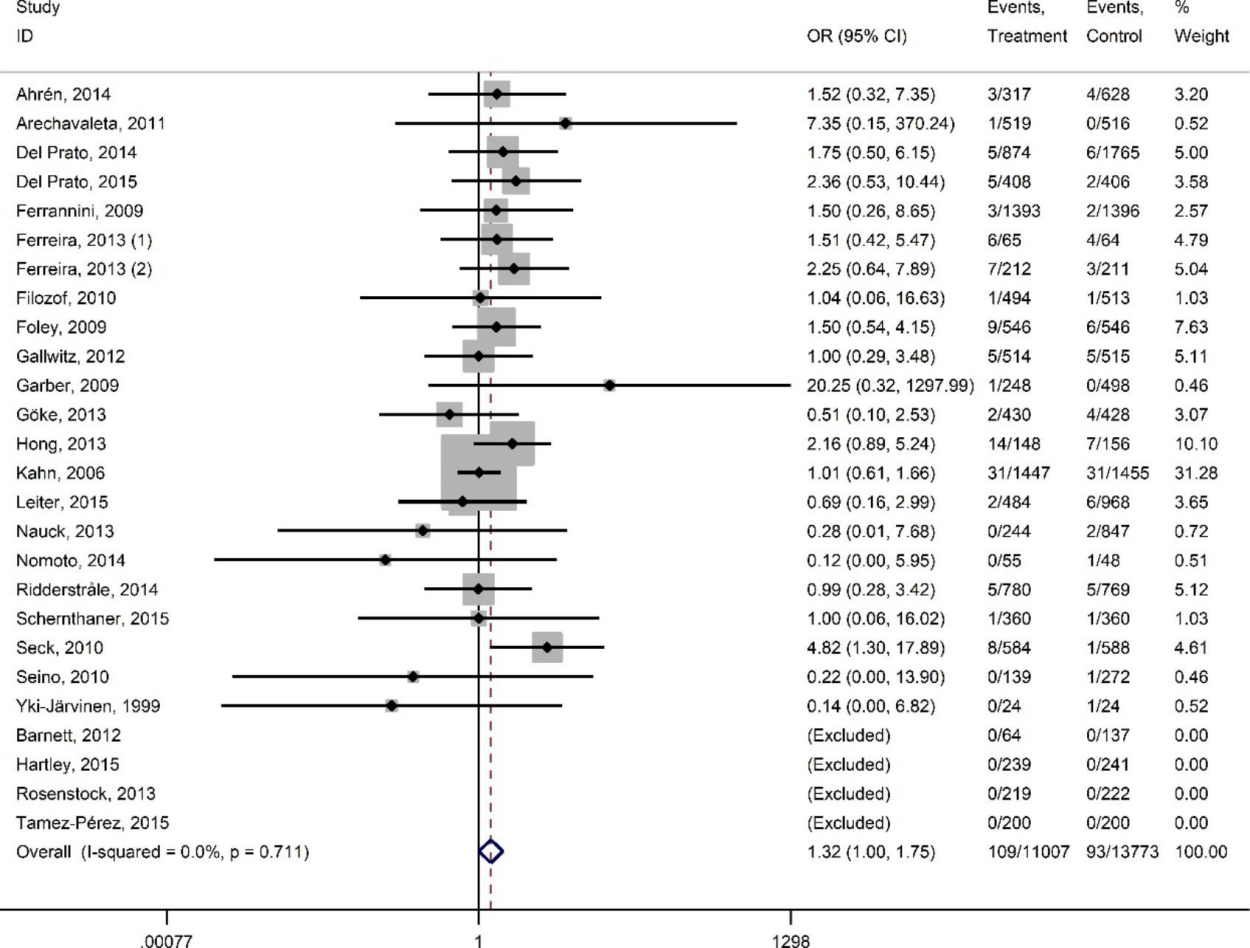
All-cause mortality of sulfonylureas versus active control. Heterogeneity chi-squared = 16.99 (d.f. = 21) p = 0.712; I^2^ = 0.0%; Test of OR = 1: z = 1.94 p = 0.053

**Fig. 3:**
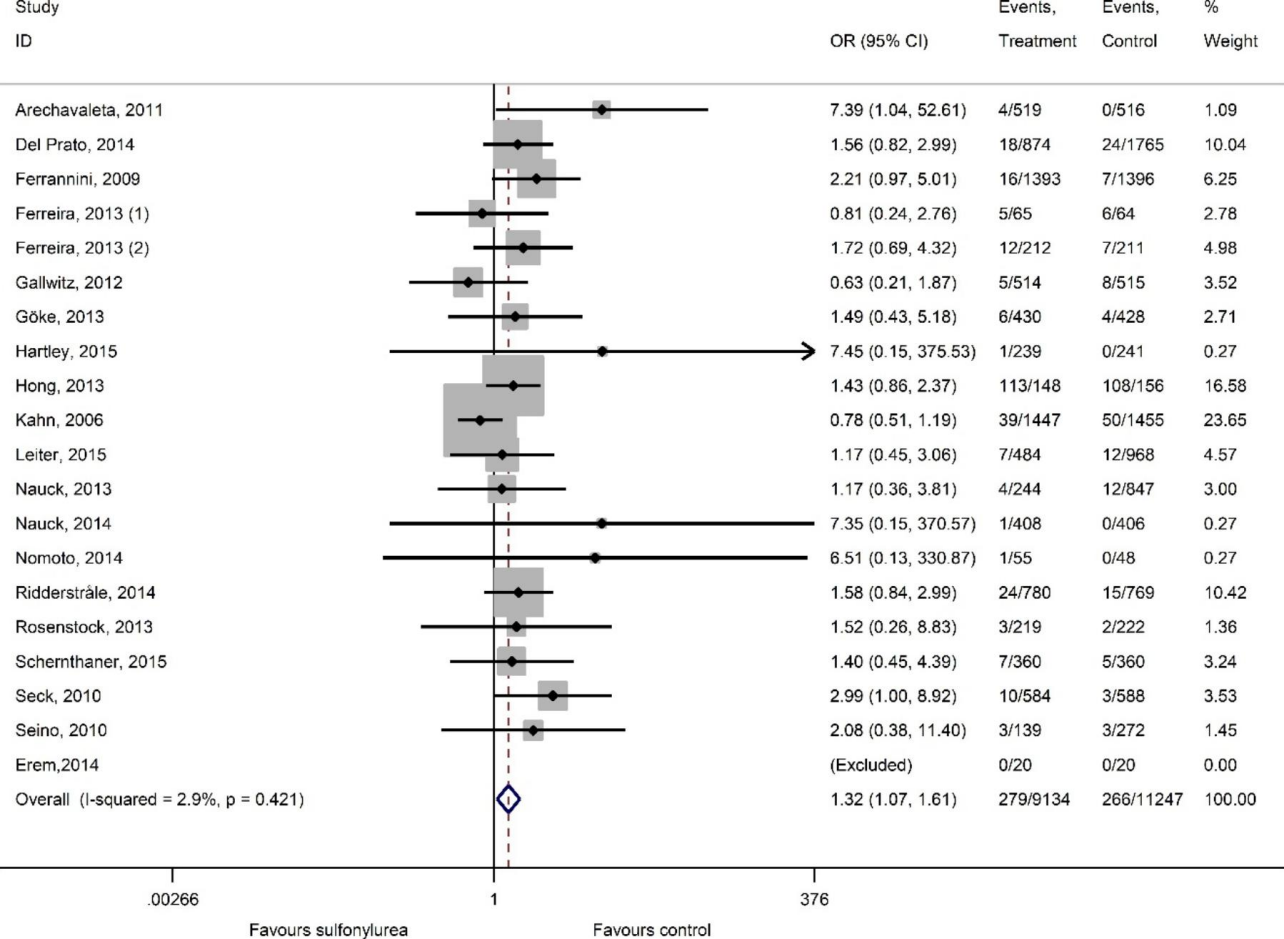
MACE, sulfonylureas versus active control. Heterogeneity chi-squared = 18.53 (d.f. = 18) p = 0.421; I^2^ = 2.9%; Test of OR = 1: z = 2.61 p = 0.009

Figures 2 and 3 present summary of findings all-cause mortality and MACE.

Compared to active control drugs with low hypoglycaemic potential (metformin, DPP-4 inhibitors, SGLT-2 inhibitors, and GLP-1 agonists), there is warning sign that sulfonylureas may increase the risk for all-cause mortality (26 studies, 24,780 participants; OR 1.32, 95% CI 1.00-1.75; I^2^ = 0%) (Fig. 2). and increased the risk for MACE (19 studies, 20,381 participants; OR 1.32, 95% CI 1.07–1.61; I^2^ = 2.9%) (Fig. 3). We evaluated the certainty of evidence with the GRADEpro tool[18] and both of the outcomes have moderate certainty.

We tested the effect of study quality on main outcome. A sensitivity analysis demonstrated that including the data from studies with low RoB only documented higher risk for all-cause mortality and MACE among those on sulfonylureas (all-cause mortality, 13 studies, 10,198 participants; OR 1.79, 95% CI 1.12–2.86, I^2^ = 0%; MACE, 8 studies, 9135 participants; OR 1.71, 95% CI 1.28–2.29, I^2^ = 0%) (Supplementary Figs. 3 and 4).

### Type of sulfonylurea

**Fig. 4:**
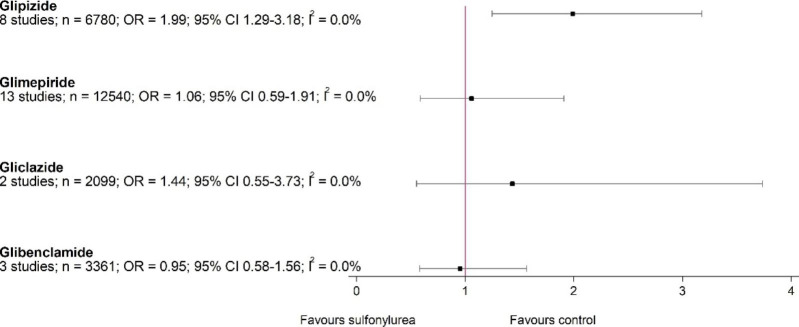
All-cause mortality of different sulfonylureas versus active control

Our subgroup analysis suggested that glipizide had the strongest effect on increasing all-cause mortality (8 studies, 6780 participants; OR 1.99, 95% CI 1.25–3.18; I^2^ = 0%), while glimepiride (13 studies, 12540 participants; OR 1.06, 95% CI 0.59–1.91; I^2^ = 0%), glibenclamide (three studies, 3361 participants; OR 0.95, 95% CI 0.58–1.56; I^2^ = 0%), and gliclazide (two studies, 2099 participants; OR 1.44, 95% CI 0.55–3.74; I^2^ = 0%) did not increase all-cause mortality (Fig. 4). We also analysed all-cause mortality according to different comparator drug classes (Supplementary Fig. 5). Sulfonylureas posed higher risk of mortality only in comparison with DPP-4 inhibitors (12 studies, 12,594 participants; OR 1.70, 95% CI 1.08–2.69; I^2^ = 0%) but not with other drug classes. However, in vast majority of studies (8 of 12 contributing 84% weight to analysis) gliclazide was used as SU.

**Fig. 5:**
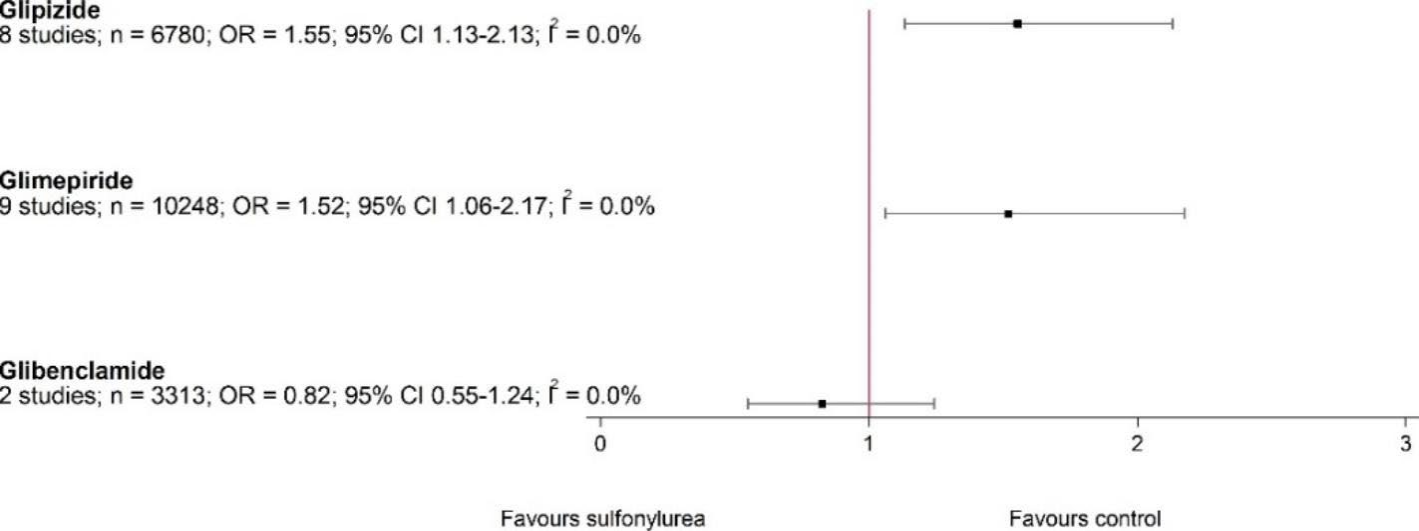
MACE, different sulfonylureas versus active control

Glipizide (8 studies, 6780 participants; OR 1.55, 95% CI 1.13–2.13; I^2^ = 0%) and glimepiride (9 studies, 10,248 participants; OR 1.52, 95% CI 1.06–2.17; I^2^ = 0%) were associated with an increased risk for MACE, while glibenclamide (2 studies, 3313 participants; OR 0.82, 95% CI 0.55–1.24; I^2^ = 0%) lacked this potential (Fig. 5).

### MACE components

**Fig. 6:**
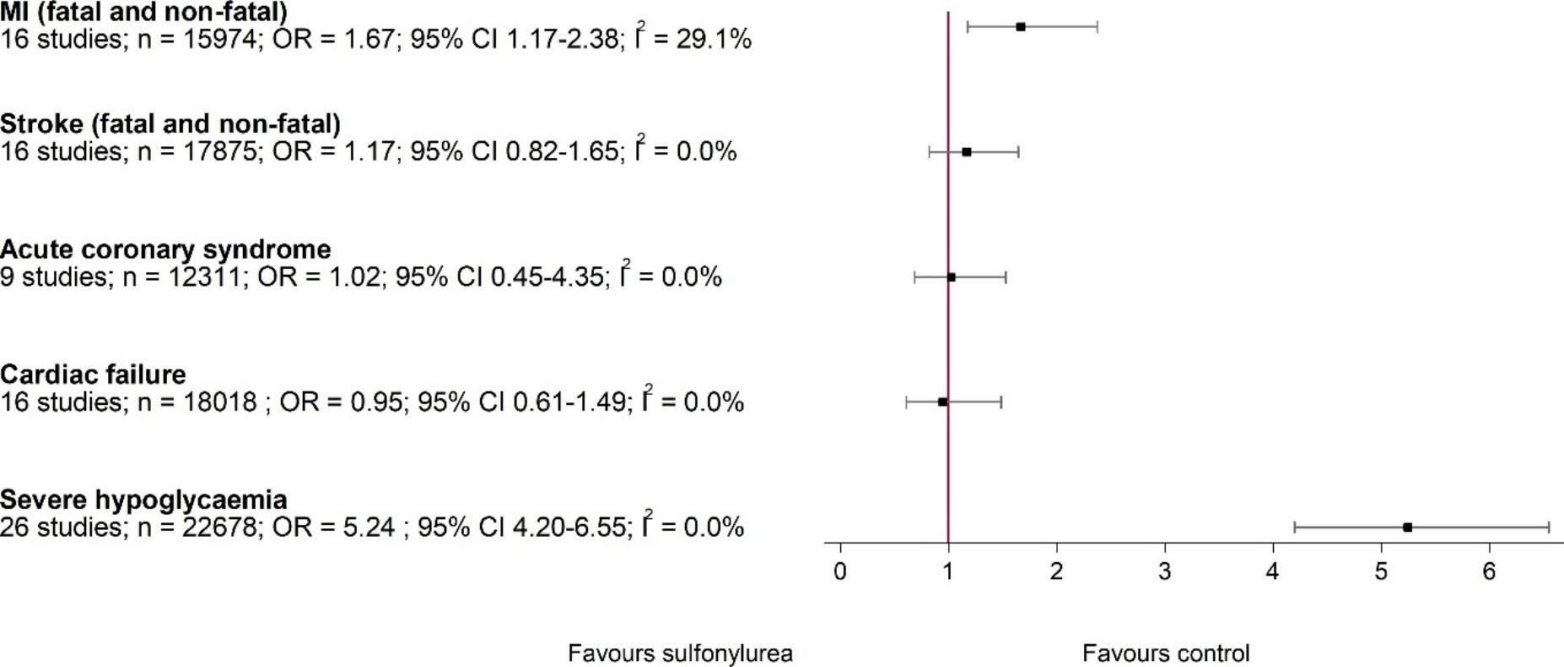
Components of MACE and severe hypoglycaemia: sulfonylureas versus active control

Figure 6 provides additional analysis by selected diseases groups. Compared to active control drugs with low hypoglycaemic potential, sulfonylureas increased the risk myocardial infarction (fatal and non-fatal) (16 studies, 15,974 participants; OR 1.67, 95% CI 1.17–2.38; I^2^ = 0%) (Fig. 6), but not stroke (fatal and non-fatal), acute coronary syndrome or cardiac failure. The effect of sulfonylureas leading to severe hypoglycaemia was strong (OR 5.24, 95% CI 4.20–6.55).

## Discussion

Evidence concerning the safety of sulfonylureas in diabetes is limited. In our analysis, including only head-to-head trials comparing SUs with drugs not inducing hypoglycaemia, we found that it is likely that the use of SUs is associated with 30% higher risk of all-cause mortality. The signal of potential harm was stronger when we limited our analysis to the studies with low risk of bias. Other meta-analyses have usually found a shift towards increased mortality with sulfonylureas in a similar range, but with variable statistical significance [4, 9]. In subgroup analysis, glipizide, but not other molecules, was associated with significantly higher all-cause mortality. A similar finding was reported at the subgroup analysis in a recent meta-analysis [10]. It is difficult to explain why glipizide differs from other analysed SU molecules (glimepiride, gliclazide, and glibenclamide). All studies included used the long-acting formulation of glipizide, so one can exclude pharmacokinetics as a reason. A recent network meta-analyses indicated that gliclazide or glimepiride use was associated with a lower mortality risk compared to that with glibenclamide, while glipizide was equal to glibenclamide [22]. In line with our results concerning glimepiride, a recent trial reported no difference in all-cause mortality between glimepiride and linagliptin in a high-risk population [23]. Collectively, distinct SU molecules may pose different risks to all-cause mortality. As the evidence is somewhat conflicting, further studies are needed to clarify the safety of distinct SUs. Meanwhile, glimepiride seems to have the best established safety background.

We also stratified the analysis of all-cause mortality by comparator drug class. SUs were associated with higher mortality only in comparison with DPP-4 inhibitors but not with other drug classes. This finding is surprising as the collective trial evidence so far has demonstrated that DPP-4 inhibitors have neutral effect on mortality and SGLT-2 inhibitors and GLP-1 RAs may have mortality benefit [24].

However, almost all DPP-4 inhibitor trials had used gliclazide and thus, the effect seems to be caused by overrepresentation of gliclazide in these trials. The prevailing concern with sulfonylureas has been cardiovascular safety. There is a potential pathophysiological link of SUs interfering with the myocardial ATP-sensitive potassium channels and affecting adaptation to myocardial ischemia [25]. The head-to-head trials using SUs have generally been small and have generated mixed evidence. In a large trial of glycaemic durability comparing rosiglitazone, metformin and glibenclamide, the sulfonylurea group had the lowest number of cardiovascular events [26]. However, it is fair to mention that the study included diabetic patients in the early stage of disease and the event rate was very low. Importantly, there is a trial with high-risk patients where use of SUs was associated with a significantly increased composite of cardiovascular endpoints [7]. As clinical trials have been focused on efficacy, they generally report cardiovascular events scarcely. As a result, it may be difficult to prove or reject the concerns about cardiovascular safety. Analysis of MACE revealed that SU use was associated with a 30% increase in the risk of MACE (p < 0.01). From the different outcomes studied, only MI (fatal or non-fatal) was increased in SU users. In the subgroup analysis, glipizide and glimepiride were associated with significantly higher numbers of cardiovascular events. Thus, in our analysis, the results of all-cause mortality and MACE clearly diverged in the case of glimepiride. Moreover, a very recent dedicated cardiovascular safety trial comparing glimepiride and linagliptin (a DPP-4 inhibitor) found no difference in the risk of MACE or number of myocardial infarctions [23]. This discrepancy probably reflects the limitations of meta-analysis in reliably detecting rare adverse effects.

Not surprisingly, SU use was associated with a 5-fold higher risk of severe hypoglycaemia.

This meta-analysis should be considered in the context of the following limitations that may affect the confidence of our findings. First, our search strategy did not include grey literature and the search period ended at 2015. We did not test the effects of setting, stage of disorder, and country of origin. It should be considered that other covariates, such as age and BMI, might influence these associations. However, statistical heterogeneity across studies was very low, which does not limit the generalizability of our conclusions. Excluding several studies due to our restrictive inclusion criteria reduced the number of studies for this meta-analysis. Clearly, relatively small numbers of events in both all-cause mortality and MACE analyses is a limitation. Moreover, we did not assess for the possible dose-response of the SUs and were unable to account for the time on-treatment. On the other hand, focusing our analysis only on studies with an active comparator were included, and thus, equal glycaemic control between study arms avoided differences arising from different glucose values. Last but not least, all-cause mortality is a strong endpoint and difficult to misreport in a well-conducted clinical trial.

## Conclusion

In comparison to drugs with low hypoglycaemic potential, sulfonylureas had higher odds for all-cause mortality, MACE, myocardial infarction (fatal and non-fatal) and hypoglycaemia. Taken together, our meta-analysis raises concerns about the possible harm related to SU therapy compared to drugs with low hypoglycaemic propensity and further studies are necessary. Important differences may exist within the class, and glimepiride seems to be the safest option according to collective evidence.

## Electronic supplementary material

Below is the link to the electronic supplementary material.

Supplementary Material 1

Supplementary Material 2

Supplementary Material 3

## Data Availability

The template data collection form is includes as Appendix 2. The data that support the findings of this study are available from the corresponding author, VV, upon reasonable request.

## Abbreviations

BMI: body mass index
CI: confidence interval
DPP-4: dipeptidyl peptidase-4
GLP-1 RA: glucagon-like peptide-1 receptor agonist
MACE: major adverse cardiovascular event
OR: odds ratio
RCT: randomized controlled trial
SGLT-2: sodium-glucose co-transporter-2
SU: sulfonylurea
