# Supplementary material for: Systematic review and meta-analysis of head-to-head trials comparing sulfonylureas and low hypoglycaemic risk antidiabetic drugs
Source: BMC Endocr Disord. 2022 Oct 19;22:251. doi: 10.1186/s12902-022-01158-5 (PMC9580135; doi:10.1186/s12902-022-01158-5)
Supplement: Supplementary file 1 — Supplementary Material 1 [file 12902_2022_1158_MOESM1_ESM.docx]

**Appendix 1.**

**Ovid MEDLINE(R) In-Process & Other Non-Indexed Citations, Ovid MEDLINE(R) Daily and Ovid MEDLINE(R)**1946 to Present

(randomized controlled trial.pt. OR controlled clinical trial.pt. OR randomized.ab.OR placebo.ab. OR clinical trials as topic.sh. OR randomly.ab. OR trial.ti. ) NOT (exp animals/not humans.sh.)

| [# ▲](http://ovidsp.tx.ovid.com.libaccess.lib.mcmaster.ca/sp-3.16.0a/ovidweb.cgi?&S=LDLOFPGOKPDDFGIJNCKKLADCEHDEAA00&Sort+Sets=descending) | Searches | Results |
| --- | --- | --- |
| 1 | (sulfonylureas or glimepiride or glyburide or glibenclamide or carbutamide or tolazamide or tolbutamide or gliclazide or glipizide or chlorpropamide or gliquidone or acetohexamide).mp. [mp = title, abstract, original title, name of substance word, subject heading word, keyword heading word, protocol supplementary concept word, rare disease supplementary concept word, unique identifier] | 20765 |
| 2 | (diabet or diabete*).mp. [mp = title, abstract, original title, name of substance word, subject heading word, keyword heading word, protocol supplementary concept word, rare disease supplementary concept word, unique identifier] | 447524 |
| 3 | 1 and 2 | 8551 |
| 4 | limit 3 to yr = "2012-Current" | 1247 |
| 5 | ((randomized controlled trial or controlled clinical trial).pt. or randomized.ab. or placebo.ab. or clinical trials as topic.sh. or randomly.ab. or trial.ti.) not (exp animals/not humans.sh.) | 895613 |
| 6 | 4 and 5 | 296 |
| 7 | exp Sulfonylurea Compounds/ | 16531 |
| 8 | exp Diabetes Mellitus/ | 330934 |
| 9 | 7 and 8 | 6918 |
| 10 | 5 and 9 | 793 |
| 11 | limit 10 to yr = "2012-Current" | 151 |
| **12** | **6 or 11** | **365** |
