# Supplementary material for: Systematic review and meta-analysis of head-to-head trials comparing sulfonylureas and low hypoglycaemic risk antidiabetic drugs
Source: BMC Endocr Disord. 2022 Oct 19;22:251. doi: 10.1186/s12902-022-01158-5 (PMC9580135; doi:10.1186/s12902-022-01158-5)

**Suppl. Figure 1. Risk of bias**

**Suppl. Figure 2 Risk of bias of the different domains**

**Suppl. Figure 3 All-cause mortality of sulfonylureas versus active control in studies with low risk of bias**

**Suppl. Figure 4 MACE of sulfonylureas versus active control in studies with low risk of bias**

**Suppl. Figure 5. All-cause mortality of different control groups versus sulfonylureas**


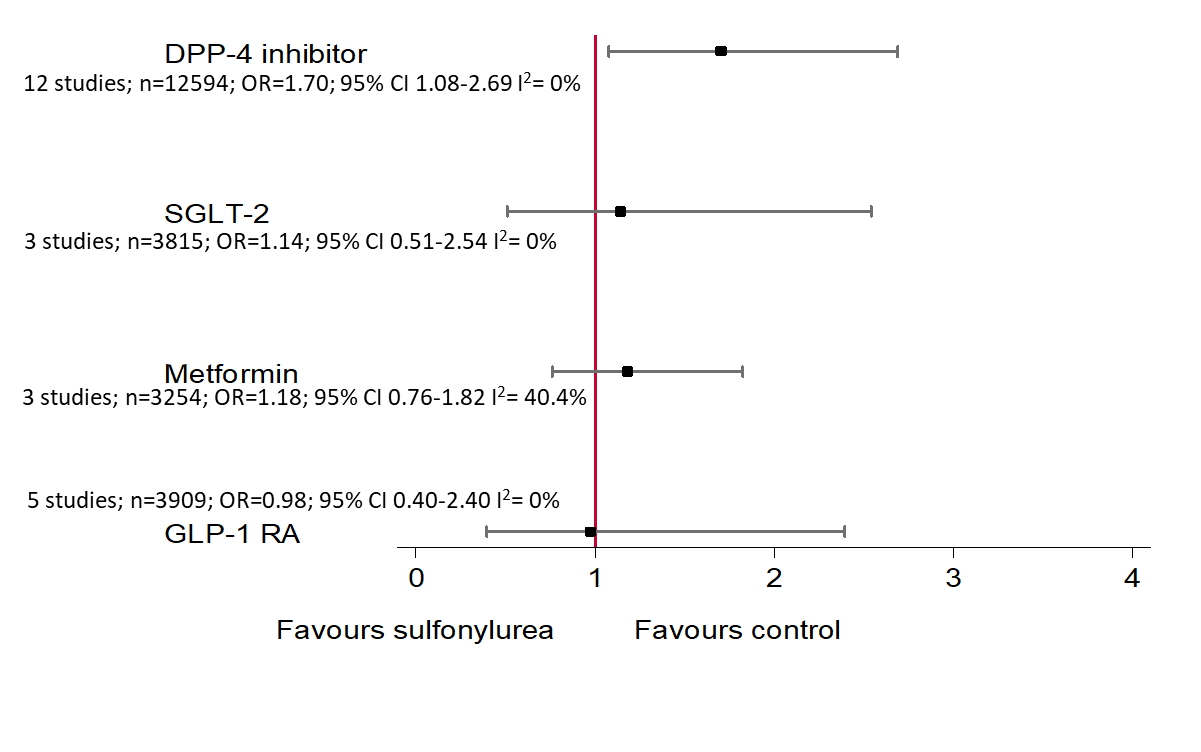


**Suppl. Figure 6 Funnel plot for all-cause mortality**


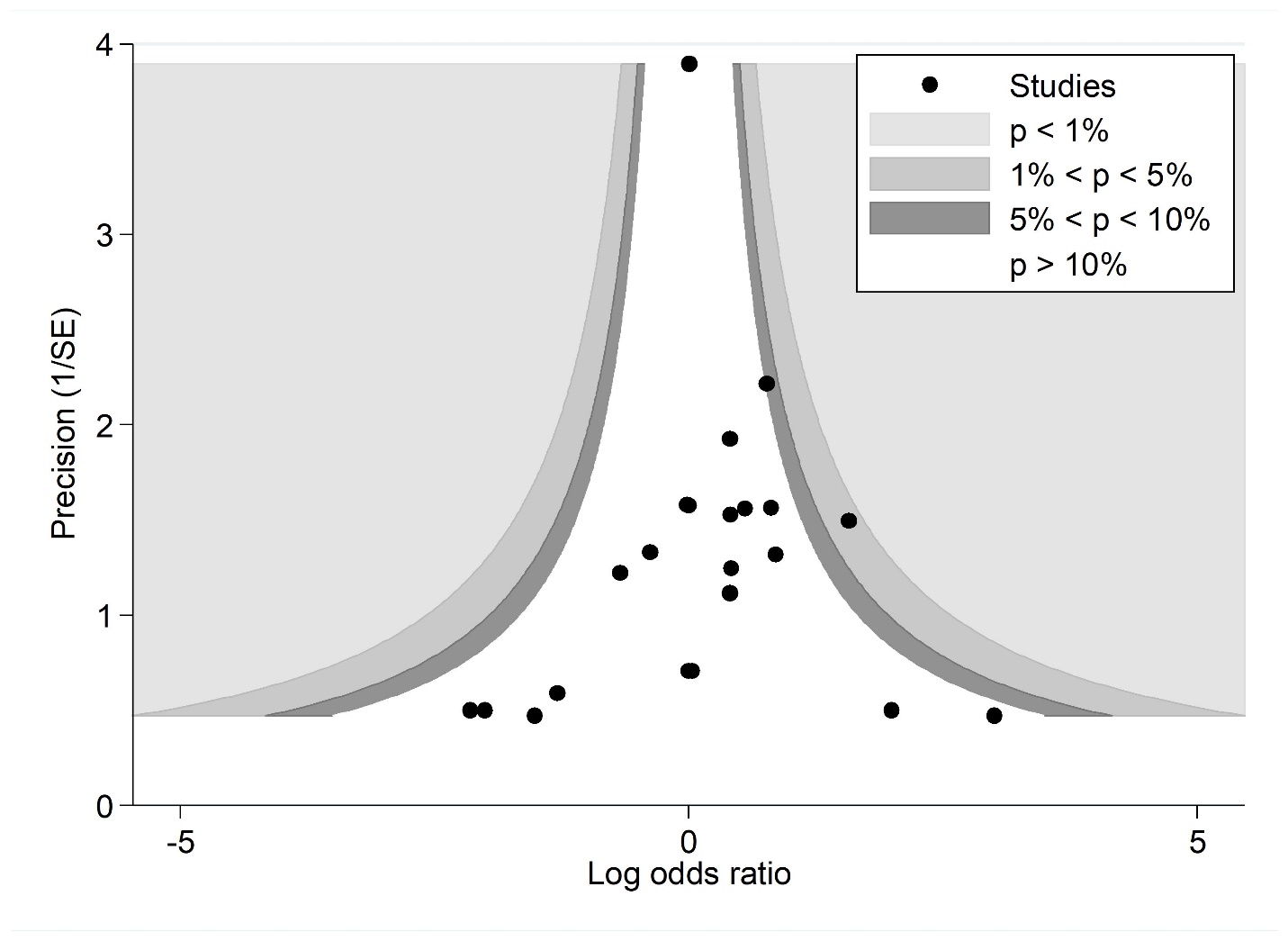


**Suppl. Figure 7** **Funnel plot for major cardiovascular events**


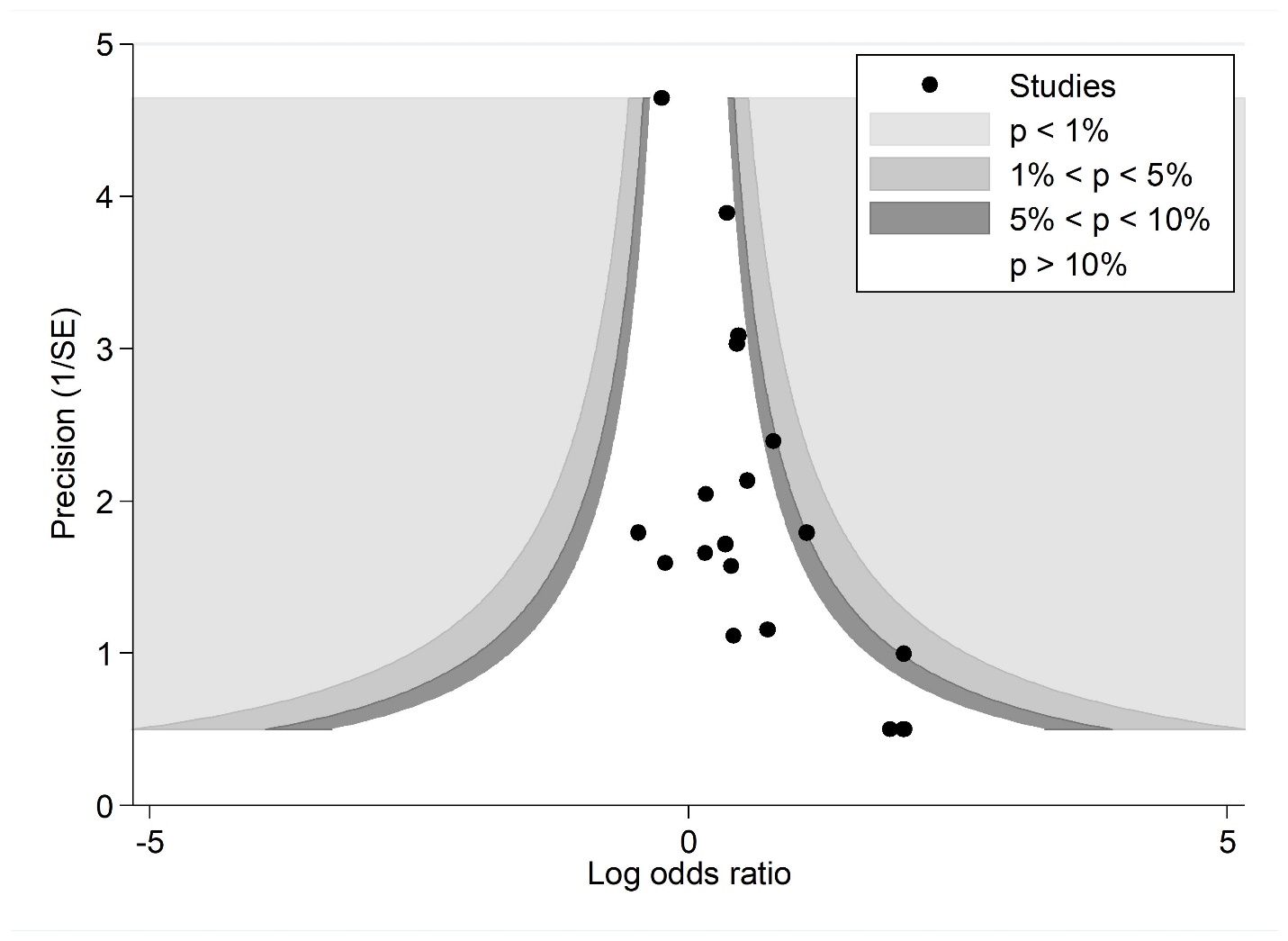

Supplement: Supplementary file 2 — Supplementary Material 2 [file 12902_2022_1158_MOESM2_ESM.docx]
