# Supplementary material for: Systematic review and meta-analysis of head-to-head trials comparing sulfonylureas and low hypoglycaemic risk antidiabetic drugs
Source: BMC Endocr Disord. 2022 Oct 19;22:251. doi: 10.1186/s12902-022-01158-5 (PMC9580135; doi:10.1186/s12902-022-01158-5)
Supplement: Supplementary file 3 — Supplementary Material 3 [file 12902_2022_1158_MOESM3_ESM.pdf]

TITLE

1. Source

**Study ID** (author, date)	
**Report ID** (unique number)	
**Other reports of this study**? list IDs	
**Reviewer ID**	
**Date of extraction** (dd/mm/yyyy)	
**Study author contact details**	

2.1. Eligibility

**Study characteristics**	**Eligibility criteria**	**Eligibility criteria met?** Yes/ No / Unclear	**Location in text or source** (page, figure, table, other)
**Type of study**	Randomised controlled trial		
**Disease**	Type 2 diabetes		
**Participants**	Women and men aged over 18, in any setting [non pregnant]		
**Types of intervention**	Sulphonylurea - monotherapy or combination therapy		
**Types of comparison**	Active control drug with low hypoglycemic potential (metformin, DPP-4 inhibitor, SGLT-2 inhibitor, GLP-1 agonist)		
**Duration of follow up**	At least 24 weeks		
**Should this trial be included in the review?**<br><br>**YES or NO**<br>**If NO give reasons:**			

2.2. Additional eligibility information [for eligible studies]

		Yes/ No / Unclear	Location in text or source (page, figure, table. other)
**Adverse effects described**	Yes/No/Only generic statement provided (**)		
**Methods of adverse effects data collection**	1- prospective/routine monitoring,<br>2- spontaneous reporting,<br>3- patient checklist/questionnaire/diary;<br>4- systematic survey of patients<br>5- unclear	Specify, with the authors' text from the paper/source	
**Primary outcomes reported (any)**	Yes/No/Only generic statement provided		
**Primary outcomes reported Number**	Yes/No		
**0**	Major adverse cardiovascular events (MACE)		
**1**	Cardiovascular mortality		
**2**	Cardiac mortality		
**3**	Fatal MI		
**4**	Nonfatal MI		
**5**	Fatal stroke		
**6**	Nonfatal stroke		
**7**	Acute coronary syndrome		
**8**	Heart failure (reported as serious)		
**9**	All cause mortality		
**10**	Severe hypoglycaemia		
**Clinical endpoints reported (any)**	Yes/No		
**11**	HcA1c		
**12**	BMI		
**13**	Body mass		
**COMMENTS**			

PROCEED ONLY IF PRIMARY OUTCOMES [MACE, CARDIOVASCULAR ADVERSE EVENTS, ALL CAUSE MORTALITY, SEVERE HYPOGLYCAEMIA] WERE REPORTED.

3. General information

Authors	
Title	
Journal	
Vol; issue; page	
Date of publication	
Link to full text	
Study author contact details	

4. Study methods

	**Descriptions as stated in report/paper**	**Location in text or source** (page, table, figure)
**Design**		
**Start date of enrollment**		
**End date of enrollment**		
**Duration of participation** (as reported by authors) (from recruitment to last follow-up for an individual person e.g. 24 weeks)		
**Ethical approval obtained for study**		
**Informed consent obtained for study**		
**Principle of data analysis** Intention to treat / per protocol / as treated / other / unclear		

5. Risk of bias assessment for primary outcomes [MACE, CARDIOVASCULAR ADVERSE EVENTS, ALL CAUSE MORTALITY, SEVERE HYPOGLYCAEMIA]

Domain	Risk of bias<br>Low<br>High<br>Unclear	Support for judgement<br>(include direct quotes where available with explanatory comments)<br>Who has been blinded?
**Random sequence generation** (selection bias)		
**Allocation concealment** (selection bias)		
**Blinding of participants and personnel** (performance bias)		
**Blinding of outcome assessment** (detection bias) Outcomes 1-10		
**Blinding of outcome assessment** (detection bias) , endpoints		
**Incomplete outcome data** (attrition bias) Outcomes 1-10		
**Incomplete outcome data** (attrition bias) endpoints		
**Is there a protocol available?**<br>**If yes - provide link**		

**Clinical trial registration number**<br><br>**If yes - provide link**		
**Selective outcome reporting?**<br>(reporting bias)		
**Comments**		

6. Participants (total study population; as detailed as possible; if not reported - unclear)

	**Descriptions as stated in report/paper**<br>add location in text or source (page, table, figure) if needed
**Country (or region)**	
**Setting**<br>rural/urban setting; primary care / specialty clinic; teaching / non hospital; else [specify]; unclear	
**Inclusion criteria**	
**Exclusion criteria**	
**Method of recruitment of participants**<br>(e.g. phone, mail, clinic patients)	
**Procedures**	
**Adherence assessment**<br>**Yes (specify if used) /No**	
**Follow-up**<br>(duration in time units reported)	
**Total number assessed for eligibility**	
**Total number excluded and reasons**	
**Total number randomised**	
**Baseline imbalances**<br>(no/yes (specify)/unclear)	

Record the following data as reported by authors; if data on all / total study population is not provided report data by study groups (see next paragraph)	
**Age (years)**<br>(mean (SD); lower and upper age limits)	
**Gender**<br>(% male)	
**Co-morbidities**<br>(no/yes/unclear)	
If co-morbities – YES – describe<br>History CV disease<br>History heart failure	
**Co-medications**<br>(no/yes/unclear)	
If co-medication – YES – describe<br>**Not taking** OAD	
**T2D treatment naïve / non naïve**<br>Yes / No (specify) / Unclear	
**Duration of T2D**<br>(duration in time units reported)	
**Subgroups mentioned**<br>(in methods section or protocol)<br>(no/yes/unclear)	
**Subgroups reported**<br>(no/yes/unclear)	
**Other relevant socio-demographic data**<br>(no/yes (specify) /unclear)	

7. Interventions

ONLY COLLECT DATA ON COMPARISON GROUPS OF INTEREST (SEE S2.1)

(cut and paste these tables as many times as necessary)

**Total number of groups**	
**Groups treated equally?**<br>(other than the intervention)<br>(no/yes/unclear)	
If unequal treatment – YES - describe	

**Intervention group 1**<br>**SULPHONYLUREA**<br>[NAME OF THE DRUG]	
MONO OR COMBINATION THERAPY	
If combination therapy, specify concomitant T2D drug(s):<br>drug, drug preparation, route of administration, dose, and frequency	
**Number randomised to Intervention group 1**	
**Description of intervention as stated in report/paper**<br>add location in text or source (page, table, figure)	
**Co-interventions**<br>(no/yes/unclear)	
**Withdrawals and exclusions**<br>(by study group on follow up = after randomization to end of study) [specify]	

**Intervention group 2**<br>**ACTIVE CONTROL**<br>[NAME OF THE DRUG]	
MONO OR COMBINATION THERAPY	
If combination therapy, specify concomitant T2D drug(s):<br>drug, drug preparation, route of administration, dose, and frequency	
**Number randomised to Intervention group 2**	
**Description of intervention as stated in report/paper**<br>add location in text or source (page, table, figure)	

**Co-interventions**<br>(no/yes/unclear)	
**Withdrawals and exclusions**<br>(by study group on follow up = after randomization to end of study) [specify]	

8. Outcomes/endpoints

(cut and paste these tables as many times as necessary)

8.0 - Major adverse cardiovascular events (MACE)

	**Descriptions as stated in report/paper**
**Time points reported**	
End of study (yes / no)	
**Outcome/endpoint definition**<br>Reported by authors (specify) / Not reported	
**Person measuring/reporting**<br>Reported (specify) / Not reported	

8.1 - Cardiovascular mortality

	**Descriptions as stated in report/paper**
**Time points reported**	
End of study (yes / no)	
**Outcome/endpoint definition**<br>Reported by authors (specify) / Not reported	
**Person measuring/reporting**<br>Reported (specify) / Not reported	

8.2 - Cardiac mortality

	**Descriptions as stated in report/paper**
**Time points reported**	
End of study (yes / no)	
**Outcome/endpoint definition**<br>Reported by authors (specify) / Not reported	
**Person measuring/reporting**<br>Reported (specify) / Not reported	

8.3 - Fatal MI

	**Descriptions as stated in report/paper**
**Time points reported**	
End of study (yes / no)	
**Outcome/endpoint definition**<br>Reported by authors (specify) / Not reported	
**Person measuring/reporting**<br>Reported (specify) / Not reported	

8.4 - Nonfatal MI

	**Descriptions as stated in report/paper**
**Time points reported**	
End of study (yes / no)	
**Outcome/endpoint definition**<br>Reported by authors (specify) / Not reported	
**Person measuring/reporting**<br>Reported (specify) / Not reported	

8.5 - Fatal stroke

	**Descriptions as stated in report/paper**
**Time points reported**	
End of study (yes / no)	
**Outcome/endpoint definition**<br>Reported by authors (specify) / Not reported	
**Person measuring/reporting**<br>Reported (specify) / Not reported	

8.6 - Nonfatal stroke

	**Descriptions as stated in report/paper**
**Time points reported**	
End of study (yes / no)	
**Outcome/endpoint definition**<br>Reported by authors (specify) / Not reported	
**Person measuring/reporting**<br>Reported (specify) / Not reported	

8.7 - Acute coronary syndrome

	Descriptions as stated in report/paper
**Time points reported**	
End of study (yes / no)	
**Outcome/endpoint definition** Reported by authors (specify) / Not reported	
**Person measuring/reporting** Reported (specify) / Not reported	

8.8 - Heart failure (reported as serious)

	Descriptions as stated in report/paper
**Time points reported**	
End of study (yes / no)	
**Outcome/endpoint definition** Reported by authors (specify) / Not reported	
**Person measuring/reporting** Reported (specify) / Not reported	

8.9 - All cause mortality

	Descriptions as stated in report/paper
**Time points reported**	
End of study (yes / no)	
**Outcome/endpoint definition** Reported by authors (specify) / Not reported	
**Person measuring/reporting** Reported (specify) / Not reported	

8.x – Other (specify) (copy as many time as needed).

	Descriptions as stated in report/paper
**Time points reported**	
End of study (yes / no)	
Other Time point(s)	
**Outcome/endpoint definition** Reported by authors (specify) / Not reported	
**Person measuring/reporting** Reported (specify) / Not reported	

8.10 - Severe hypoglycaemia

	Descriptions as stated in report/paper
**Time points reported**	
Baseline (yes / no)	
End of study (yes / no)	
**Outcome/endpoint definition**<br>Reported by authors (specify) / Not reported	
**Person measuring/reporting**<br>Reported (specify) / Not reported	

8.11 - HcA1c

	Descriptions as stated in report/paper
**Time points reported**	
Baseline (yes / no)	
End of study (yes / no)	
**Outcome/endpoint definition**<br>Reported by authors (specify) / Not reported	
**Person measuring/reporting**<br>Reported (specify) / Not reported	
**Unit of measurement or scale**	

8.12 - BMI

	Descriptions as stated in report/paper
**Time points reported**	
Baseline (yes / no)	
End of study (yes / no)	
**Outcome/endpoint definition**<br>Reported by authors (specify) / Not reported	
**Person measuring/reporting**<br>Reported (specify) / Not reported	
**Unit of measurement or scale**	

8.13 - Body mass

	Descriptions as stated in report/paper
**Time points reported**	
Baseline (yes / no)	
End of study (yes / no)	
**Outcome/endpoint definition**<br>Reported by authors (specify) / Not reported	

**Person measuring/reporting**<br>Reported (specify) / Not reported	
**Unit of measurement or scale**	

9. Results [CLINICAL OUTCOMES/ENDPOINTS]

9.0 –9.8, 9.10 Clinical outcomes

Subgroup analysis?<br>0= no, whole group analysed<br>1= yes, specify					
**End time point**<br>[in months]					
**Withdrawals [different from those described in Section 7] occurred**<br>0= no<br>1= yes, if yes specify		0			
		**Intervention group 1 (**	**Intervention group 2 (**		
**Number allocated to this group or subgroup**					
**Number receiving intervention**					
**Number completing study**					
**Number who dropped out**					
**Number of events** e.g. number of cases detected					
**0**	Major adverse cardiovascular events (MACE)				
**1**	Cardiovascular mortality				
**2**	Cardiac mortality				
**3**	Fatal MI				
**4**	Nonfatal MI				
**5**	Fatal stroke				
**6**	Nonfatal stroke				
**7**	Acute coronary syndrome				
**8**	Heart failure (reported as serious)				
10	Severe hypoglycaemia				
	Comments				

9.0 –9.8, 9.10 Clinical outcomes (Data only from ClinicalTrials.gov)

Subgroup analysis?	

0= no, whole group analysed<br>1= yes, specify				
**End time point**<br>[in months]				
**Withdrawals [different from those described in Section 7] occurred**<br>0= no<br>1= yes, if yes specify				
	**Intervention group 1 (**	**Intervention group 2)**		
**Number allocated to this group or subgroup**				
**Number receiving intervention**				
**Number completing study**				
**Number who dropped out**				

Number of events e.g. number of cases detected

**0**	Major adverse cardiovascular events (MACE)				
**1**	Cardiovascular mortality				
**2**	Cardiac mortality				
**3**	Fatal MI				
**4**	Nonfatal MI				
**5**	Fatal stroke				
**6**	Nonfatal stroke				
**7**	Acute coronary syndrome				
**8**	Heart failure (reported as serious)				
10	Severe hypoglycaemia				
	**Angina unstable**				
	**Atrial fibrillation**				
	**Cardiac Failure Chronic**				
	Comments	•			

9.9 - All cause mortality

**Subgroup analysis?**<br>0= no, whole group analysed<br>1= yes, specify	
**End time point**<br>[in months]	
**Withdrawals [different from those described in Section 7] occurred**<br>0= no	

1= yes, if yes specify				
	**Intervention group 1 (**	**Intervention group 2 ()**		
**Number allocated to this group or subgroup**				
**Number receiving intervention**				
**Number completing study**				
**Number who dropped out**				
**Number of events** e.g. number of cases detected				
**Deaths (all cause)**				
**Causes of death** (by study group if reported)				

9.X Use this table if for outcomes other than counts (case numbers) are used

(copy as many time as needed)

**Subgroup analysis?**<br>0= no, whole group analysed<br>1= yes, specify				
**End time point**<br>[in months]				
**Withdrawals [different from those described in Section 7] occurred**<br>0= no<br>1= yes, if yes specify				
	**Intervention group 1**	**Intervention group 2**	**Intervention group 3**	**Intervention group 4**
**Number allocated to this group or subgroup**				
**Number receiving intervention**				
**Number completing study**				
**Number who dropped out**				
**Number of events**<br>e.g. number of cases detected				
**OR**				
**BASELINE**				
**Number of participants analysed**				
**%**				
**95% CI**				
**SE**				
**END OF STUDY**				
**Number of participants analysed**				
**%**				
**95% CI**				

**SE**				
**OR**				
**Absolute risk difference/reduction, unadjusted**				
**95% CI**				
**SE**				
**Group with baseline risk (minuend)**				
**Group the risk is subtracted (subtrahend)**				
**Absolute risk difference/reduction, adjusted**				
**95% CI**				
**SE**				
**Variables adjusted for**				
**Group with baseline risk (minuend)**				
**Group the risk is subtracted (subtrahend)**				
**OR**				
**Risk ratio (relative risk) , unadjusted**				
**95% CI**				
**SE**				
**Group in numerator**				
**Group in denominator**				
**Risk ratio (relative risk) , adjusted**				
**95% CI**				
**SE**				
**Variables adjusted for**				
**Group in numerator**				
**Group in denominator**				
**OR**				
**Odds ratio , unadjusted**				
**95% CI**				
**SE**				
**Group in numerator**				
**Group in denominator**				
**Odds ratio , adjusted**				
**95% CI**				
**SE**				
**Variables adjusted for**				
**Group in numerator**				
**Group in denominator**				
**OR**				
**Hazard rate ratio, unadjusted**				
**95% CI**				
**SE**				
**Group in numerator**				
**Group in denominator**				

**Hazard rate ratio, adjusted**				
**95% CI**				
**SE**				
**Variables adjusted for**				
**Group in numerator**				
**Group in denominator**				

9.11 - HcA1c

**Variable type**<br>1= continuous<br>2= categorized	1
**Categories (if categorized)**	
**Subgroup analysis?**<br>0= no, whole group analysed<br>1= yes, specify	
**End time point**<br>[in months]	
**Withdrawals [different from those described in Section 7] occurred**<br>0= no<br>1= yes, if yes specify	

	**Intervention group 1**	**Intervention group 2**		
**Number allocated to this group or subgroup**				
**Number receiving intervention**				
**Number completing study**				
**Number who dropped out**				
**BASELINE**				
Number of participants analysed				
Mean				
95% CI				
**SD**				
**Range**				
**END OF STUDY**				
Number of participants analysed				
Mean				
95% CI				
SE				
**OR**				

**Least squares (LS) mean change (within group), adjusted**				
**Variables adjusted for**				
**Difference of means, unadjusted**				
**95% CI**				
**SE**				
**Group subtracted from (minuend)**				
**Group subtracted (subtrahend)**				
**Difference of LS means (between-group), adjusted**				
**95% CI**				
**SE**				
**Variables adjusted for**				
**OR**				
**Absolute risk difference/reduction, unadjusted**				
**95% CI**				
**SE**				
**Category the risk of which is compared**				
**Group with baseline risk (minuend)**				
**Group the risk is subtracted (subtrahend)**				
**Absolute risk difference/reduction, adjusted**				
**95% CI**				
**SE**				
**Variables adjusted for**				
**OR**				
**Risk ratio (relative risk) , unadjusted**				
**95% CI**				
**SE**				
**Category the risk of which is compared**				
**Group in numerator**				
**Group in denominator**				
**Risk ratio (relative risk) , adjusted**				
**95% CI**				
**SE**				
**Variables adjusted for**				
**OR**				
**Odds ratio , unadjusted**				
**95% CI**				
**SE**				
**Category the risk of which is compared**				
**Group in numerator**				
**Group in denominator**				

**Odds ratio , adjusted**				
**95% CI**				
**SE**				
**Variables adjusted for**				
**OR**				
**Hazard rate ratio, unadjusted**				
**95% CI**				
**SE**				
**Category the risk of which is compared**				
**Group in numerator**				
**Group in denominator**				
**Hazard rate ratio, adjusted**				
**95% CI**				
**SE**				
**Variables adjusted for**				

9.12 - BMI

**Variable type**<br>1= continuous<br>2= categorized	1
**Categories (if categorized)**	
**Subgroup analysis?**<br>0= no, whole group analysed<br>1= yes, specify	
**End time point**<br>[in months]	
**Withdrawals [different from those described in Section 7] occurred**<br>0= no<br>1= yes, if yes specify	

	**Intervention group 1)**	**Intervention group 2)**	**Intervention group 3**	**Intervention group 4**
**Number allocated to this group or subgroup**				
**Number receiving intervention**				
**Number completing study**				
**Number who dropped out**				
**BASELINE**				
Number of participants analysed				
Mean				
95% CI				
SD				

**END OF STUDY**				
Number of participants analysed				
Mean				
95% CI				
SE				
**OR**				
**Difference of means, unadjusted**				
**95% CI**				
**SE**				
**Group subtracted from (minuend)**				
**Group subtracted (subtrahend)**				
**Difference of means, adjusted**				
**95% CI**				
**SE**				
**Variables adjusted for**				
**OR**				
**Absolute risk difference/reduction, unadjusted**				
**95% CI**				
**SE**				
**Category the risk of which is compared**				
**Group with baseline risk (minuend)**				
**Group the risk is subtracted (subtrahend)**				
**Absolute risk difference/reduction, adjusted**				
**95% CI**				
**SE**				
**Variables adjusted for**				
**OR**				
**Risk ratio (relative risk) , unadjusted**				
**95% CI**				
**SE**				
**Category the risk of which is compared**				
**Group in numerator**				
**Group in denominator**				
**Risk ratio (relative risk) , adjusted**				
**95% CI**				
**SE**				
**Variables adjusted for**				
**OR**				
**Odds ratio , unadjusted**				
**95% CI**				
**SE**				
**Category the risk of which is compared**				

**Group in numerator**				
**Group in denominator**				
**Odds ratio , adjusted**				
**95% CI**				
**SE**				
**Variables adjusted for**				
**OR**				
**Hazard rate ratio, unadjusted**				
**95% CI**				
**SE**				
**Category the risk of which is compared**				
**Group in numerator**				
**Group in denominator**				
**Hazard rate ratio, adjusted**				
**95% CI**				
**SE**				
**Variables adjusted for**				

9.13 - Body mass

**Variable type**<br>1= continuous<br>2= categorized	
**Categories (if categorized)**	
**Subgroup analysis?**<br>0= no, whole group analysed<br>1= yes, specify	
**End time point**<br>[in months]	
**Withdrawals [different from those described in Section 7] occurred**<br>0= no<br>1= yes, if yes specify	

	**Intervention group 1**	**Intervention group 2)**		
**Number allocated to this group or subgroup**				
**Number receiving intervention**				
**Number completing study**				
**Number who dropped out**				
**BASELINE**				
Number of participants analysed				
Mean				

95% CI				
SD				
**END OF STUDY**				
Number of participants analysed				
Mean				
95% CI				
SE				
**OR**				
**Least squares (LS) mean change (within group), adjusted**				
**Difference of means, unadjusted**				
**95% CI**				
**SE**				
**Group subtracted from (minuend)**				
**Group subtracted (subtrahend)**				
**Difference of LS means (between-group), adjusted**				
**95% CI**				
**SE**				
**Variables adjusted for**				
**OR**				
**Absolute risk difference/reduction, unadjusted**				
**95% CI**				
**SE**				
**Category the risk of which is compared**				
**Group with baseline risk (minuend)**				
**Group the risk is subtracted (subtrahend)**				
**Absolute risk difference/reduction, adjusted**				
**95% CI**				
**SE**				
**Variables adjusted for**				
**OR**				
**Risk ratio (relative risk) , unadjusted**				
**95% CI**				
**SE**				
**Category the risk of which is compared**				
**Group in numerator**				
**Group in denominator**				
**Risk ratio (relative risk) , adjusted**				
**95% CI**				
**SE**				

**Variables adjusted for**				
**OR**				
**Odds ratio , unadjusted**				
**95% CI**				
**SE**				
**Category the risk of which is compared**				
**Group in numerator**				
**Group in denominator**				
**Odds ratio , adjusted**				
**95% CI**				
**SE**				
**Variables adjusted for**				
**OR**				
**Hazard rate ratio, unadjusted**				
**95% CI**				
**SE**				
**Category the risk of which is compared**				
**Group in numerator**				
**Group in denominator**				
**Hazard rate ratio, adjusted**				
**95% CI**				
**SE**				
**Variables adjusted for**				

9.xx. Use this form id endpoint data is categorized

(copy as many times as needed)

**Variable type**<br>1= continuous<br>2= categorized				
**Categories (if categorized)**				
**Subgroup analysis?**<br>0= no, whole group analysed<br>1= yes, specify				
**End time point**<br>[in months]				
**Withdrawals [different from those described in Section 7] occurred**<br>0= no<br>1= yes, if yes specify				
	**Intervention group 1**	**Intervention group 2**	**Intervention group 3**	**Intervention group 4**
**Number allocated to this group or subgroup**				

**Number receiving intervention**				
**Number completing study**				
**Number who dropped out**				
**Number of persons by categories (if categorized)**<br>Category 1<br>Category 2<br>etc				
**OR**				
**BASELINE**				
**Number of participants analysed**				
**Mean if continuous, % in categories if categorized**<br>Category 1<br>Category 2<br>etc				
**95% CI**				
**SE**				
**END OF STUDY**				
**Number of participants analysed**				
**Mean if continuous, % in categories if categorized**<br>Category 1<br>Category 2<br>etc				
**95% CI**				
**SE**				
**OR**				
**Difference of means, unadjusted**				
**95% CI**				
**SE**				
**Group subtracted from (minuend)**				
**Group subtracted (subtrahend)**				
**Difference of means, adjusted**				
**95% CI**				
**SE**				
**Variables adjusted for**				
**OR**				
**Absolute risk difference/reduction, unadjusted**				
**95% CI**				
**SE**				
**Category the risk of which is compared**				
**Group with baseline risk (minuend)**				
**Group the risk is subtracted (subtrahend)**				

**Absolute risk difference/reduction, adjusted**				
**95% CI**				
**SE**				
**Variables adjusted for**				
**OR**				
**Risk ratio (relative risk) , unadjusted**				
**95% CI**				
**SE**				
**Category the risk of which is compared**				
**Group in numerator**				
**Group in denominator**				
**Risk ratio (relative risk) , adjusted**				
**95% CI**				
**SE**				
**Variables adjusted for**				
**OR**				
**Odds ratio , unadjusted**				
**95% CI**				
**SE**				
**Category the risk of which is compared**				
**Group in numerator**				
**Group in denominator**				
**Odds ratio , adjusted**				
**95% CI**				
**SE**				
**Variables adjusted for**				
**OR**				
**Hazard rate ratio, unadjusted**				
**95% CI**				
**SE**				
**Category the risk of which is compared**				
**Group in numerator**				
**Group in denominator**				
**Hazard rate ratio, adjusted**				
**95% CI**				
**SE**				
**Variables adjusted for**				

10. Miscellaneous

**Funding source declared**<br>Yes / No<br>IF reported: specify	

Government / Industry / else …	
**Key conclusions from study authors:**	
**Comments from study authors on adverse effects:**	
**Correspondence required and outcome of correspondence:**<br>Yes / No	
**References identified:**	
**Comments from reviewer:**	
